# Central Line Bundle Implementation in US Intensive Care Units and Impact on Bloodstream Infections

**DOI:** 10.1371/journal.pone.0015452

**Published:** 2011-01-18

**Authors:** E. Yoko Furuya, Andrew Dick, Eli N. Perencevich, Monika Pogorzelska, Donald Goldmann, Patricia W. Stone

**Affiliations:** 1 Division of Infectious Diseases, Columbia University College of Physicians and Surgeons, New York, New York, United States of America; 2 RAND Corporation, Boston, Massachusetts, United States of America; 3 Department of Internal Medicine, Iowa City VA and University of Iowa Carver College of Medicine, Iowa City, Iowa, United States of America; 4 Columbia University School of Nursing, New York, New York, United States of America; 5 Institute for Healthcare Improvement, Cambridge, Massachusetts, United States of America; 6 Children's Hospital Boston, Boston, Massachusetts, United States of America; 7 Columbia University School of Nursing, New York, New York, United States of America; National Institute of Allergy and Infectious Diseases, National Institutes of Health, United States of America

## Abstract

**Background:**

Central line-associated bloodstream infections (CLABSI) represent a serious patient safety issue. To prevent these infections, bundled interventions are increasingly recommended. We examine the extent of adoption of Central Line (CL) Bundle elements throughout US intensive care units (ICU) and determine their effectiveness in preventing CLABSIs.

**Methodology/Principal Findings:**

In this cross-sectional study, National Healthcare Safety Network (NHSN) hospitals provided the following: ICU-specific NHSN-reported rates of CLABSI/1,000 central line days; policies and compliance rates regarding bundle components; and other setting characteristics. In 250 hospitals the mean CLABSI rate was 2.1 per 1000 central line days and 49% reported having a written CL Bundle policy. However, of those that monitored compliance, only 38% reported very high compliance with the CL Bundle. Only when an ICU had a policy, monitored compliance, *and* had ≥95% compliance did CLABSI rates decrease. Complying with any one of three CL Bundle elements resulted in decreased CLABSI rates (β = -1.029, p = 0.015). If an ICU without good bundle compliance achieved high compliance with any one bundle element, we estimated that its CLABSI rate would decrease by 38%.

**Conclusions/Significance:**

In NHSN hospitals across the US, the CL Bundle is associated with lower infection rates only when compliance is high. Hospitals must target improving bundle implementation and compliance as opposed to simply instituting policies.

## Introduction

Healthcare-associated infections (HAIs) are a major source of morbidity and mortality despite often being preventable. Most HAIs are associated with an invasive device and occur in intensive care units (ICUs). Of an estimated 99,000 HAI-related deaths per year, bloodstream infections lead to an estimated 31,000 deaths per year, with a mean attributable cost of $18,000 per central line-associated bloodstream infection (CLABSI).[1], [2]

In partnership with other national and scientific organizations, the Institute for Healthcare Improvement (IHI) promoted “care bundles” as part of its effort to improve patient safety.[3] The IHI Central Line (CL) Bundle consists of five interventions: hand hygiene; maximal barrier precautions; chlorhexidine skin antisepsis; optimal catheter site selection, with avoidance of the femoral vein for central venous access in adult patients; and daily review of the line necessity, with prompt removal of unnecessary lines.[4]

This bundle is being widely promoted for implementation across the country. For example, 28 state hospital associations have joined collaboratives to reduce CLABSI in part by using the CL Bundle.[5] Additionally, as part of the National Patient Safety Goals (NPSG), beginning in 2010, The Joint Commission is requiring inclusion of the use of a checklist based on the CL Bundle (NPSG.07.04.01 items 11-12, 14-17, 17).

Despite the promotion of bundles, questions remain about their adoption and effectiveness. Bundled policies do not guarantee reliable execution at the bedside; moreover, even though decreases in CLABSIs have been reported,[6] these infections continue to be significant problems in many ICUs.[7] Quasi-experimental studies point to subsequent decreases in CLABSI rates following bundle implementation.[8], [9] In these publications, a key focus has been improving the culture of safety, and some have hypothesized that an overall heightened attention to one clinical issue leads to a positive “chain reaction” effect that prevents other complications.[10] If this were the case, implementing one bundle would be expected to lead to a decrease in other non-targeted HAI rates in the same setting. In this study, we examine the extent of adoption of Central Line (CL) Bundle elements throughout US intensive care units (ICU) and determine their effectiveness in preventing CLABSIs.

## Methods

### Objectives

The objectives of this study were to: 1) examine the extent of adoption of the CL Bundle in ICUs across the US; 2) determine the effectiveness of individual bundle elements on reducing infections; and 3) determine the effectiveness of combinations of the bundle elements on reducing infections; and 4) determine if the effect of the bundle elements was specific, or if compliance reduced infection rates in non-targeted HAIs such as ventilator-associated pneumonia (VAP).

### Participants

To participate, a hospital must have conducted National Healthcare Safety Network hospitals (NHSN) CLABSI surveillance in an adult medical, medical/surgical, or surgical ICU in 2007 according to CDC protocol;[11] and the ICU must have had a minimum of 500 device days. There were 441 hospitals eligible to participate. Our NHSN expert (TH) developed a list of eligible hospitals and invited them to participate.

### Survey

The survey, described in detail elsewhere, was conducted as part of a larger study assessing infection control resources and practices and was thus designed to be answered by the director or manager of each hospital's infection control department.[12] Respondents were asked about ICU-specific policies and practices related to the CL in eligible ICUs. Because we hypothesized that having policies alone was insufficient to decrease infection rates, for each bundle component, respondents were asked: a) whether the ICU had a written CL Bundle policy in place; b) whether compliance was monitored; and c) if so, how often compliance was observed (all of the time/95% - 100%; usually/75% - 94%, sometimes/25% - 74%, rarely or never/<25%; and don't know). Because compliance with hand hygiene affects all HAIs, compliance with this crucial policy was not considered specific to the bundle; rather, it was measured in another section of the survey as a setting-specific characteristic.

Respondents were also asked about the number of years of NHSN/NNIS (i.e., National Nosocomial Infection Surveillance system, the precursor to NHSN) membership. State mandatory HAI reporting requirements were identified. Other setting characteristics included hospital teaching status, size and ICU type.

### Ethics

All procedures were reviewed and approved by institutional review boards (IRB) at Columbia University, CDC, and RAND Corporation. The requirement to obtain written documentation of informed consent was waived by the IRBs in accordance with 45 C.F.R. § 46.117(c). An online information sheet explaining the study was provided to each of the participants.

### Outcome Measurement

Hospitals reported ICU-specific quarterly CLABSI rates as reported to the NHSN. In collecting these data, all facilities follow a specific surveillance protocol that defines CLABSIs using standard CDC definitions.[11] This protocol developed by CDC epidemiologists include accurate case finding and has both laboratory and clinical criteria,[13], [14] and the protocol has become the recognized standard for CLABSI identification globally.[15], [16] The sensitivity and specificity of this protocol has been reported to be 85% and 98.3% respectively.[17]

### Statistical Analysis

Descriptive statistics were computed. To understand the generalizability of our sample, we compared the hospital demographics and CLABSI rates to published data on all NHSN hospitals for the same year.

Individual bundle components were characterized based on compliance during the last time it was monitored. If the rate of compliance was missing (versus respondent indicating “don't know”), we assumed compliance to be low and set the value as rarely/never.

We evaluated the CL Bundle in two ways: by including and excluding the chlorhexidine skin antisepsis element. We did this because, while the use of chlorhexidine is easily implemented and there has been strong evidence for its effectiveness at reducing CLABSI in many instances,[18] there has also been evidence of its decreased activity against certain pathogens (gram-negative and fungal microorganisms)[19] as well as an association between resistance to methicillin and chlorhexidine.[20]-[22]

To meet our aims, we first explored if simply having a policy in place, monitoring compliance with the policy, and/or the level of compliance was associated with a reduction in CLABSI. Once that needed level of compliance was established, we then conducted a set of separate multivariate analyses. In the first analysis, we examined the impact of the individual bundle elements (Model 1). Model 2 tested the effect of compliance with at least one element versus compliance with no elements. Model 3 tested the effect of compliance with all bundle elements versus compliance with no or some elements. Last, to help with the translation of these findings, we calculated the expected percent reduction of CLABSI if an ICU was able to increase compliance from just one bundle elements to two elements.

Finally, we wished to assess the validity of our data in order to exclude any bias due to self-reporting. That is, we wanted to further exclude the possibility that certain hospitals may have over-reported CL Bundle compliance and underreported CLABSI rates in order to make themselves look better than they really were. To exclude this possibility, in each model we examined the CL Bundle's “cross-over” effects by assessing whether compliance with the CL Bundle was associated with lower rates of ventilator-associated pneumonia (VAP). As part of the survey we thus asked hospitals to report their ICU-specific quarterly VAP rates as reported to NHSN.

All models were multivariate ordinary least squares regressions with Huber-White standard errors to account for intra-hospital correlation across ICUs.[23] We controlled for setting characteristics including ICU type, hand hygiene compliance, and other hospital characteristics such as region, bed size and teaching status as described above. Lastly, based on the multivariate results we predicted how infection rates would change if an ICU moved from no bundle implementation to full compliance.

## Results

### Descriptives

A total of 250 hospitals participated (response rate 57%). Table 1 describes the hospitals and ICUs in the study sample. Hospitals in our sample tended to be on the larger end of the NHSN spectrum; only 68.4% of our hospitals had 500 beds or less as compared to 84.2% of NHSN hospitals.[24] This is consistent with our eligibility criteria, which required that hospitals have at least 500 device days in order to participate in the survey. The northeast region contributed the largest number of study hospitals (109 or 44%). Just over 75% of the hospitals (189) were from states with mandatory HAI reporting requirements. Most hospitals (54%) had participated in the NHSN/NNIS for more than three years.

**Table 1:** Description of hospitals and ICUs.

**Hospital Characteristics (N = 250)**		
**Region**	N	%
Northeast	109	44
South	66	26
Midwest	40	16
West	35	14
**Mandatory Reporting (State)**	189	76
**Bed Count**		
<201	50	20
201 - 500	145	58
501 - 1000	50	20
>1000	5	2
**Hand Hygiene Compliance (N = 240)**		
All of the time (95-100%)	17	7
Usually (75-94%)	104	43
Sometimes (25-74%)	79	33
Rarely/never (<25%)	1	0.4
**ICU Type (N = 415)**	N	%
Medical	103	25
Medical/Surgical	223	54
Surgical	89	21

CL Bundle data were available from 415 ICUs, as some hospitals provided data on more than one ICU. Only 312 of the ICUs reported CLABSI rates and the mean rate was 2.1/1,000 line days (standard deviation [sd] = 2.8, range 0 to 22). Table 2 provides a breakdown of the CLABSI rates by ICU type in our sample and in all NHSN hospitals. The study sample's infection rates were similar to all NHSN hospitals, suggesting that participants were not dissimilar from the average NHSN hospital.

**Table 2:** Comparison of CLABSI rates by ICU Type in the study sample to all participating ICUs in the NHSN.

	Study Sample			NHSN		
	N	Pooled mean (SD)	Median	N	Pooled mean	Median
**CLABSI**						
Medical ICU	74	2.6 (3.2)	1.7	144	2.4	1.9
Medical/Surgical Teaching	111	1.7 (1.9)	1.1	104	2	1.5
Medical/Surgical Other	60	1.6 (2.8)	0	343	1.5	0.6
Surgical	67	2.6 (3.4)	2	128	2.3	1.7

CLABSI  =  Central Line-Associated Bloodstream Infections, ICU  =  Intensive Care Unit, NHSN  =  National Healthcare Safety Network.

Table 3 provides a breakdown of CL Bundle elements, including whether the policy was in place, whether it was monitored, and the extent each element was executed correctly based on the last time monitored. While 49% of ICUs had a written policy regarding the CL Bundle, only 38% of ICUs that monitored the policy (n = 35) reported having implemented the CL Bundle 95% of the time or greater. Overall, maximal barrier precautions was the most commonly implemented element, while daily line checks and optimal site selection were least commonly implemented.

**Table 3:** Extent to which the ICUs have written policies, monitor implementation and proportion of time the bundle element is correctly implemented.

	Presence of Written Policy		Presence of Monitoring for Implementation		ICUs Reporting Correct Implementation					
					*At Least Sometimes		At Least Usually		All of the Time	
	N	%	N	%	N	%	N	%	N	%
**Central Line Bundle**	204	49	91	45	70	77	62	68	35	38
Barrier precautions	392	94	292	74	247	85	242	83	186	64
Chlorhexidine use	394	95	266	68	229	86	223	84	194	73
Optimal site selection	235	57	133	57	116	87	104	78	58	44
Daily infection check	341	82	194	57	152	78	148	76	104	54

ICU  =  Intensive Care Unit.

*“At Least Sometimes” refers to ICUs with compliance “sometimes” to some bundle elements and “sometimes,” “usually,” or “all the time” to other bundle elements.

### Multivariate Analyses

#### Hospital and Infection Prevention and Control Department Characteristics

Table 4 provides the results of the multivariate analyses. No setting characteristic, including hospital size, geographic location and teaching status, had a significant impact on HAI rates. Hand hygiene compliance was not independently associated with HAI rates.

**Table 4:** Multivariate regression analysis of association between central line bundle elements and CLABSI rates controlling for setting characteristics.

	CLABSI					
	Coef	SE	P-Value	95% CI		
**Model 1: Individual Impact of Each Element**						
**CLABSI Bundle Elements**						
Barrier precautions	-0.420	0.710	0.550	-1.815	to	0.975
Chlorhexidine use	0.350	0.667	0.600	-0.961	to	1.661
Optimal site selection	-0.617	0.543	0.260	-1.684	to	0.450
Daily check	-0.706	0.430	0.100	-1.551	to	0.139
**Model 2: Impact of Complying With Any One Element**						
**CLABSI 3-Elements** *						
At least 1 element	-1.029	0.421	0.015	-1.856	to	-0.201
**Model 3: Impact of Complying With All Bundle Elements**						
**CLABSI Bundle**						
All elements	-0.318	0.943	0.736	-2.171	to	1.535

CI =  Confidence Interval, CLABSI  =  Central Line-associated Bloodstream Infections.

*CLABSI 3-Elements excluded chlorhexidine skin antisepsis from the model. Note: In all models the following co-variates were controlled for: geographic region, teaching status, hand hygiene, years in NHSN, bedsize, and type of ICU.

#### CL Bundle Policies

In multivariate analyses, we initially examined if simply having a CL Bundle policy in place led to lower CLABSI rates, but this was not the case. Similarly, monitoring compliance with a bundle policy but having less than ideal compliance (<95%) showed no association with lower CLABSI rates. Thus, we conducted our multivariate analyses looking for an association between high (≥95%) compliance and CLABSI rates.

Taking each CL Bundle element individually, there were no statistically significant associations with CLABSI rates; however, all elements trended towards lowering CLABSI rates with the exception of chlorhexidine use (Model 1). When chlorhexidine was excluded, we found that having at least one of the bundle elements implemented correctly (Model 2) decreased CLABSI rates very significantly (β = -1.029, p = 0.015). We did not find that complying with all bundle elements (as opposed to complying with no or some elements) was not necessary to show a decrease in infections (Model 3). Our results do imply that if an average ICU that complied with no components of the CL Bundle infection rate were to comply with at least one component of the bundle all the time, they would experience an estimated 38% decrease in its CLABSI rate (a reduction from an estimated rate of 2.722 to 1.694). Last, there was no convincing evidence of a cross-over effect; that is, for all models, compliance with the CL Bundle elements was never associated with a decrease in VAP rates.

## Discussion

To our knowledge, this is the first national study that examines the real-world practices of monitoring and implementing the Central Line Bundle in US ICUs, as well as measuring its impact on CLABSI rates in a detailed fashion. We found that there is wide variability in both CL Bundle compliance and infection rates. While it appears that instituting bundle policies is becoming common (a CL Bundle policy was present in almost half of our ICUs), only a disappointing 38% of those that monitored bundle implementation reported full compliance.

This is significant because we found that there was no relationship between simply having a bundle policy in place and lower infection rates. Moreover, even monitoring the bundle elements and having moderate compliance was not enough to lower CLABSI rates. We found that only when an ICU had a bundle policy, monitored compliance with it, *and* had 95% or greater compliance did CLABSI rates decrease.

Pronovost *et al.* found that participation in a collaborative study focused on actively implementing the CL Bundle lowered CLABSI rates from .62 to .34 per 1000 central line days.[8] Our study predicted that if an ICU lacking high compliance to any bundle element achieved high compliance with any one bundle element, its CLABSI rate would decrease by 38%. Pronovost also recently reported that when an ICU continues rigorous monitoring of compliance, low CLABSI rates are sustained.[25] We find that not only is monitoring needed, continuous high compliance is also crucial.

We did not find a significant difference between having high compliance with all of the bundle elements and having high compliance with at least one element. This result may be due to lack of statistical power and the few ICUs in our sample that actually complied with all elements or a lack of an incremental effect. While the care bundle is intended to represent an “all or nothing” approach, it is possible that as long as ICUs perform even one intervention meticulously, an improvement in infection rates can be achieved. Indeed, we did find that complying with any one element was effective. To our knowledge, the incremental effect of the elements in the CL Bundle has not been previously studied. More studies are needed to fully understand this finding.

The chlorhexidine element was not associated with a decrease in CLABSI rates when examined individually, and it weakened the association of the other bundle elements with CLABSI rates. While chlorhexidine has been shown to be an effective antiseptic agent in numerous studies, it is possible that it is not always being used optimally in the real world setting. For instance, it is important to allow the agent to dry fully before line insertion, and perhaps inserters are not allowing this to occur. Simply using chlorhexidine may not be enough; compliance with good technique in using it may be required. An alternative explanation may be that while it is well documented that chlorhexidine is very effective in killing gram-positive organisms such as staphylococci, its efficacy against organisms such as gram negative bacilli and fungi such as *Candida* species is more limited.[19], [26] Furthermore, it has been well described that there is an increasing trend in HAI, including CLABSI, caused by these organism types.[6] With the wide promotion of the CL Bundle across the country, including the use of chlorhexidine for skin antisepsis, future research is warranted.

### Limitations

There are a number of limitations to this study. Data were collected through a survey approach with a 57% response rate which is comparable to recent surveys of hospital personnel with reported response rates of 38-53%.[27]-[29] To examine the possibility of non-response bias we compared the HAI rates in the respondent hospitals to those found in published estimates of all NHSN hospitals. The rates were similar. Second, data were self-reported by hospitals' infection control departments' director/manager. One issue stemming from mandatory public reporting is the immense pressure for hospitals to underreport these adverse outcomes. This pressure to “look good” could motivate hospitals to underreport CLABSI rates (to NHSN and in our survey) and also to implement and/or over-report compliance with the CL Bundle, thus making the association between HAI rates and bundle strategies appear stronger than it really is. However, we controlled for mandatory reporting and this was not significantly associated with lower CLABSI rates. Also, in our data we found some hospitals reporting higher CLABSI rates also reported less compliance with the bundles.

Moreover, to more fully test our results, we performed a “cross-bundle” analysis in which we looked for an association between CL Bundle compliance and an untargeted HAI, namely rates of ventilator-associated pneumonia. If some hospitals were intent upon making themselves look better, one would assume that they would have reported CL Bundle compliance, CLABSI rates, and VAP rates as all being better than they were. In that case, high compliance with the CL Bundle would be associated both with lower VAP rates as well as lower CLABSI rates. However, we found only an association between the CL Bundle and CLABSI and no association between the CL Bundle and VAP rates. Thus, the cross-bundle analyses suggest that there is no significant bias due to self-reporting.

Finally, there is the possibility of unobserved and unmeasured factors confounding the HAI rates, such as patients' risk severity. We controlled for many known confounders, and these factors rarely affected HAI rates to any significant degree. Nonetheless, future research will include visits to these ICUs to directly observe compliance, review reported data, and assess for the presence of other confounding variables.

Last, our data were collected from larger NHSN hospitals, based on our criteria of a minimum number of device days for study eligibility. At that time, NHSN hospitals themselves were mainly large urban tertiary hospitals, thus smaller, rural institutions may have different findings.

### Conclusion

In this national study of real-world practices in US ICUs, the Central Line Bundle is frequently promoted but seldom well implemented. The new Joint Commission requirement of universal central line “checklists” incorporating the CL Bundle is unlikely to decrease CLABSI rates unless there is proper implementation and very high compliance.
